# Chiral Aspects of Local Anesthetics

**DOI:** 10.3390/molecules25122738

**Published:** 2020-06-12

**Authors:** Ružena Čižmáriková, Jozef Čižmárik, Jindra Valentová, Ladislav Habala, Mário Markuliak

**Affiliations:** 1 Department of Chemical Theory of Drugs, Faculty of Pharmacy, Comenius University, 832 32 Bratislava, Slovakia; cizmarikova@fpharm.uniba.sk (R.Č.); valentova@fpharm.uniba.sk (J.V.); markuliak@fpharm.uniba.sk (M.M.); 2 Department of Pharmaceutical Chemistry, Faculty of Pharmacy, Comenius University, 832 32 Bratislava, Slovakia; cizmarik@fpharm.uniba.sk

**Keywords:** local anesthetic, stereochemistry, chiral, pharmacology, pharmacokinetics

## Abstract

Thanks to the progress made in chemical technology (particularly in the methodologies of stereoselective syntheses and analyses) along with regulatory measures, the number of new chiral drugs registered in the form of pure enantiomers has increased over the past decade. In addition, the pharmacological and pharmacokinetic properties of the individual enantiomers of already-introduced racemic drugs are being re-examined. The use of the pure enantiomer of a drug that has been used to date in the form of a racemate is called a “chiral switch”. A re-examination of the properties of the pure enantiomers of racemates has taken place for local anesthetics, which represent a group of drugs which have long been used. Differences in (*R*) and (*S*)-enantiomers were found in terms of pharmacodynamic and pharmacokinetic activity as well as in toxicity. Levobupivacaine and robivacaine were introduced into practice as pure (*S*)-(−)-enantiomers, exhibiting more favorable properties than their (*R*)-(+)-stereoisomers or racemates. This overview focuses on the influence of chirality on the pharmacological and toxicological activity of local anesthetics as well as on individual HPLC and capillary electrophoresis (CE) methods used for enantioseparation and the pharmacokinetic study of individual local anesthetics with a chiral center.

## 1. Introduction

Local anesthetics are substances that reversibly prevent the formation and spread of the signals in the peripheral sensory nerves, i.e., through their activity, nerves are temporarily numbed. In the case of topical anesthesia, this concerns the numbing of delimited areas or tissues by using aqueous solutions, ointments, or powders. In the case of the infiltration anesthesia, larger amounts of the diluted solution are injected into the tissue of the respective area. In the case of the conduction anesthesia, a smaller quantity of concentrated solution is used to inhibit the course of the nerve trunk. Particular types of conduction anesthesia include spinal anesthesia, epidural anesthesia, etc. The effect of local anesthetics is increased or prolonged by vasoconstrictors (adrenaline, corbadrine) that slow down the backflushing of the anesthetic by blood [1]. At higher concentrations, local anesthetics can also block vegetative and motor nerve fibers. Side effects include effects on the central nervous system and cardiovascular system, which are also used therapeutically.

The general structure of local anesthetics involves lipophilic/hydrophilic parts and a linker [2,3]. According to their chemical structure they can be divided into several groups:

- Anesthetics of the ester type (procaine, tetracaine, oxyprocaine, proxymetacaine)
- Amide- and anilide-type anesthetics (cinchocaine, procaine)
- Anesthetics related to basic ketones (falicaine) and basic ethers (fomocaine)

The physicochemical properties of local anesthetics correlate with their pharmacokinetic, clinical, and toxicological properties. A selection of these properties is given in Table 1 [4,5].

**Figure 1:**
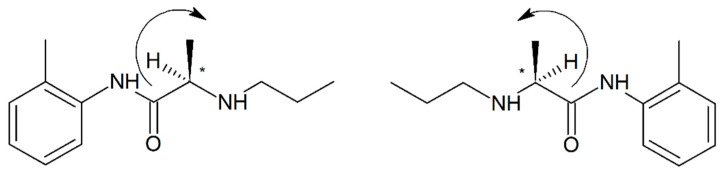
Structures and stereochemistry of (*R*)-prilocaine (left) and (*S*)-prilocaine (right).

Similar to other types of drugs [6,7,8,9,10,11,12], the activity of anesthetics is determined not solely by their structure and physicochemical properties but also by their stereochemical arrangement. In many structures of local anesthetics, especially of the amide type, the stereocenter is on the carbon atom, which causes the formation of two (*R*) and (*S*)-enantiomers which differ in their pharmacodynamic, pharmacokinetic, and toxicological properties. Pursuant to the Cahn–Ingold–Prelog system [13], the individual stereoisomers (+) and (−) have an absolute configuration of (*R*) and (*S*), respectively. An example is prilocaine (Figure 1).

Local anesthetics have been used in clinical practice for more than a century. Today there are numerous such drugs in use. The global market value for 2020 is estimated at US$ 4251.5 million [14]. The areas of application include stomatology, ophthalmology, gynaecology, and plastic surgery, among others. The main local anesthetics in use are bupivacaine, lidocaine, benzocaine, ropivacaine, prilocaine, and chloroprocaine. The most dominant drug on the market is still lidocaine, although the fastest growth in the years to come is expected for bupivacaine. The share of newer drugs such as articaine, ropivacaine, or levobupivacaine is also expected to rise. The prevailing application form is as an injectable anesthetic [15]. Besides achiral or racemic compounds, there is also an expanding market for enantiomerically pure local anesthetics. The Compound Annual Growth Rate (CAGR) for the most successful of them, levobupivacaine, is anticipated to be 3.5% from 2020 to 2027. The market value for this compound was US$840.4 million by 2019. The marketing prospects of ropivacaine, a local anesthetic distributed as a pure enantiomer, are also improving. [16].

## 2. Mechanism of the Action of Local Anesthetics

The mechanism of the activity is based on several facts. In the first hypothesis, a major role is attributed to acetylcholine, which is released in the impulse and is bound on the membrane protein receptors acting in the resting condition as an ionic barrier. This changes the structure of proteins, membrane permeability increases, and the exchange of sodium and potassium ions allows for the signal to spread [17].

Amphiphilic local anesthetics interact hydrophobically and electrostatically with lipid bilayers and modify their physicochemical properties, with direct inhibition of membrane functions. The resultant alteration of the membrane lipid environments surrounding transmembrane proteins and the subsequent protein conformational change lead to the inhibition of channel functions [18].

Another theory attaches importance to the pores of the nerve membranes. The lipophilic parts of the molecules can bind on their surface, either through the surface or their chains. Another hypothesis supposes a mutual competition of Ca^2+^ and local anesthetics in their bonding to the membrane phospholipids [19,20].

The impact of stereochemistry on the activity of anesthetics in general has been addressed in several past reviews [21,22,23]. In 2006, Tomin et al. specifically discussed the activity of local anesthetics with respect to their stereochemical arrangement [24]. The presented overview focused on local anesthetics, which, due to their different stereochemistry, have different local anesthetic activities, pharmacokinetic properties, and toxicity [25,26,27]. Emphasis was given to their pharmacological properties, including also novel developments. Enantioseparation techniques for the racemic local anesthetics are discussed as well.

## 3. Chirality in the Pharmacodynamics and Toxicity of Local Anesthetics

### 3.1. Cocaine

**Scheme 1:**
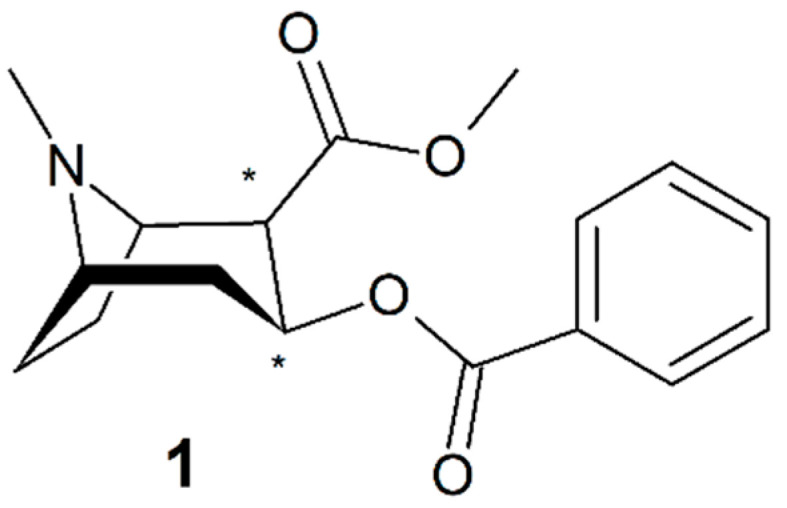
Cocaine (1).

The first local anesthetic, from which other structural types were derived, was (−)-cocaine (**1**, Scheme 1), the natural alkaloid from *Erythroxylium coca*. Racemic and (+)-cocaine can be obtained by chemical synthesis. In fact, there are only eight stereoisomers because the *N*-methyl-nitrogen bridge binds C(1) and C(5)- only in the *cis* conformation [28]. Of individual stereoisomers, (+)-cocaine showed a 2-fold higher local anesthetic activity compared to (−)-cocaine [29,30].

Toxic effects, including convulsions and lethal effects, were evaluated in mice. The levorotatory (−)-enantiomer of cocaine increased locomotor activity at doses of at least 340 times lower than the highest doses of (+)-enantiomer. Toxic effects were seen with both enantiomers of cocaine, where (−)-cocaine brought about approximately 8 to 13 times more intensive convulsions and lethality than its opposite enantiomer [31]. Cocaine may be addictive and therefore is subject to the Narcotics Act.

### 3.2. Mepivacaine

**Scheme 2:**
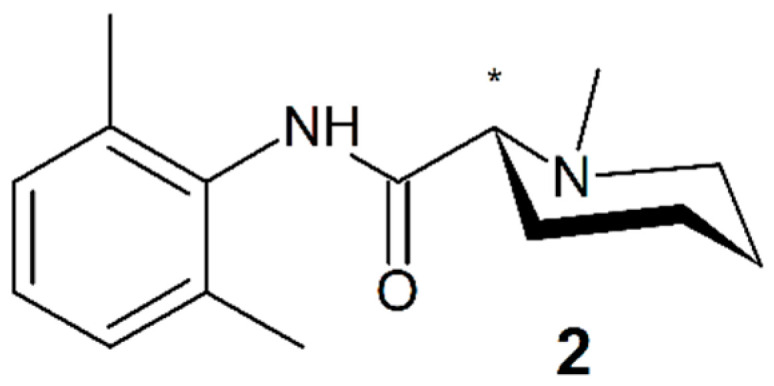
Mepivacaine (**2**).

Mepivacaine, *N*-(2,6-dimethylphenyl)-1-methylpiperidine-2-carboxamide (**2**, Scheme 2), is an amide-type local anesthetic which is used in the form of a racemic mixture in all types of infiltration anesthesia as well as in regional nerve block anesthesia. The enantiomers differ from each other in their biological activity. The (*S*)-(−) enantiomer is more biologically active in comparison with (*R*)-(+)-mepivacaine [32,33].

### 3.3. Prilocaine

**Scheme 3:**
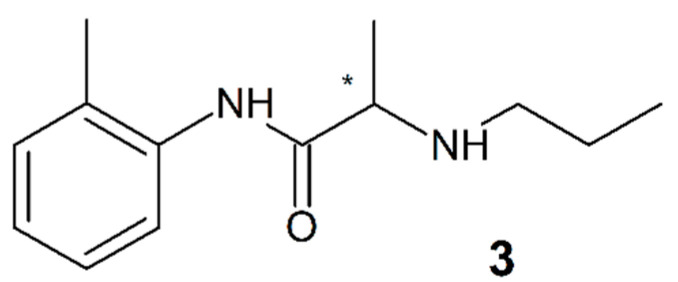
Prilocaine (**3**).

Prilocaine, *N*-(2-methylphenyl)-2-(propylamino)propanamide (**3**, Scheme 3), is another anilide-type local anesthetic used as a racemate for IV regional nerve block and topical anesthesia. Prilocaine is less effective than butacaine and both its isomers have similar local anesthetic activity in experiments on animals in vivo. Smaller differences were observed in experiments on animals in vitro. From the toxicity point of view, the (*R*)-(−)-enantiomer exhibits higher toxicity [24,34].

### 3.4. Bupivacaine and Levobupivacaine

**Scheme 4:**
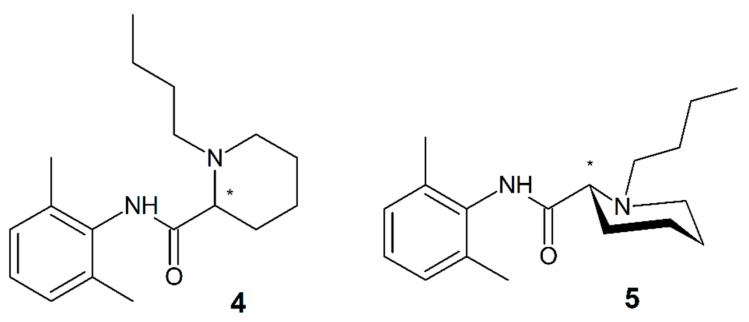
Bupivacaine (**4**) and levobupivacaine (**5**).

The local anesthetic bupivacaine, 1-butyl-*N*-(2,6-dimethylphenyl)piperidine-2-carboxamide (**4**, Scheme 4), exists in the two stereoisomeric forms of (*R*)-(+)- and (*S*)-(−)-bupivacaine. Due to their lower toxicity to the heart and central nervous system, experiments led to the introduction of (*S*)-(−)- bupivacaine–levobupivacaine (**5**, Scheme 4) into clinical anesthesia [35].

Cox et al. [36] reached the conclusion that (*S*)-(−)-bupivacaine had similar properties in extradural anesthesia as its racemate, but, as a local anesthetic in the brachial plexus block, (*S*)-(−)-bupivacaine is the preferred enantiomer [37,38]. Another study showed that (*S*)-(−)-bupivacaine exhibited, in comparison with the racemic form and the (*R*)-enantiomer, a strong vasoconstriction effect. The weakest effect was exhibited by the (*R*)-enantiomer, while the effect of racemic bupivacaine was between the two enantiomers [39].

The direct myocardial effect of (*S*)-(−)- and (*R*)-(+)-enantiomers of bupivacaine was compared in the papillary isolated muscle of guinea pigs. The recorded action potential duration (APD) was mostly shortened in the presence of (*R*)-(+)-bupivacaine across a wide range of pacing frequencies. (*S*)-(−)-bupivacaine affected APD in guinea pig papillary muscle less than the (*R*)-(+)-enantiomer at various stimulation rates and resting membrane potentials [40].

For individual stereoisomers of bupivacaine, the effect on biomimetic membranes containing cardiolipin and cholesterol was compared. The order of the individual isomers was: (*S*)-(−)- enantiomer < racemate < (*R*)-(+)-enantiomer, which correlates also with the toxic effects of these local anesthetics [41]. Nau et al. [42] confirmed that the toxic action of bupivacaine, mainly the (*R*)-(+)-isomer, may be associated with blockade of potassium channels.

(*S*)-(−)-bupivacaine has a more potent phasic blocking effect than ropivacaine or (*R*)-(+)-bupivacaine in crayfish giant axons in vitro. For the three anesthetics an equivalent intracellular local anesthetic concentration was found, indicating that the intracellular cationic local anesthetic concentration is not directly connected with the intensity of block [43]. Levobupivacaine and bupivacaine produced comparable and significantly persistent antinociceptive effects in comparison with ropivacaine at all concentrations tested after epidural and intrathecal administration [44].

The inhibitory effects of bupivacaine on the Na^+^ ion channel involve binding to the inactivated state of the Na^+^ channel and to the activated or open state; the stereoselectivity is based on the interaction with the inactivated state. The dissociation from rested or inactivated blocked states is not stereoselective [45]. Molecular modelling revealed that the enantiomers differed significantly in the position of the *N*-substituent if superimposed by their aromatic rings, which could explain the higher potency of (*R*)-(+)-bupivacaine [46]. According to the model, the (*R)-*(+) enantiomer adopts a more favorable conformation in energetic terms than the (*S)-*(−) enantiomer. The authors claimed that the aromatic ring of the drug would initially interact with an amino acid containing an aromatic ring (Phe, Tyr or Trp), forming a π‒π interaction between both aromatic rings. This interaction is more stable than the hydrophobic interaction between alkyl chains. Subsequently, the *N*-substituent would interact with the two amino acids identified as constituents of the receptor site for bupivacaine in hKv1.5 channels (Leu or Val). Thus, this interaction is stereocontrolled by the stereochemistry of the chiral carbon of the local anesthetic [46].

A comparison between racemic bupivacaine and levobupivacaine in a mixture with lignocaine for peribulbar block in cataract surgery was performed [47]. No significant differences between the two treated groups of patients were encountered with regard to the akinesia score, number of supplementary injections, and initial and total required volume of local anesthetics.

A randomized double-blinded study in spinal anesthesia was performed to evaluate the anesthetic potencies and hemodynamics of intrathecal levobupivacaine in comparison with racemic bupivacaine [48]. No significant difference between the two groups in the quality of motor and sensory block could be found. The majority of patients (60%) in the levobupivacaine group had sensory onset time between 1 and 5 min, whereas in the racemic bupivacaine group 56% of patients had a sensory onset time of 6–10 min. Nausea, vomiting, and shivering were more frequent in the racemic bupivacaine group.

### 3.5. Ropivacaine

**Scheme 5:**
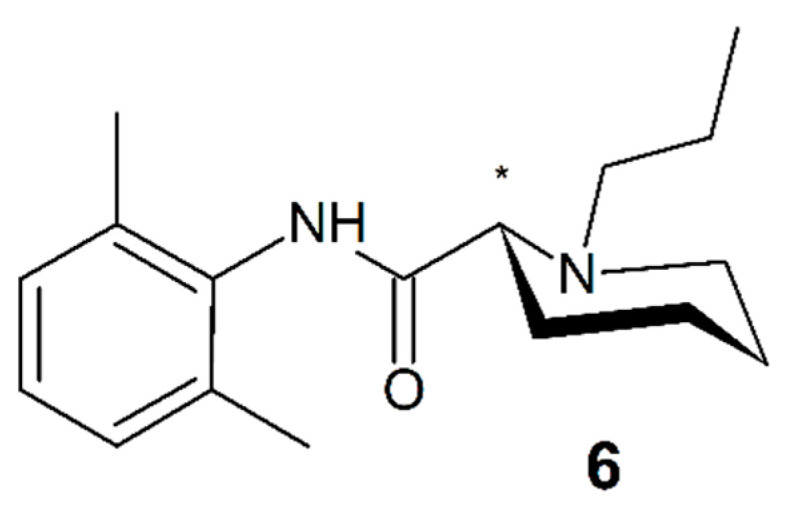
Ropivacaine (**6**).

Ropivacaine, (2*S*)-(−)-*N*-(2,6-dimethylphenyl)-1-propylpiperidine-2-carboxamide (**6**, Scheme 5), was the first local anesthetic to be used in clinical practice as a pure (*S*)-(−)-enantiomer. On the piperidine nitrogen, propyl is bonded instead of butyl, where it differs from the structurally close bupivacaine. Its pharmacodynamic and pharmacokinetic profile is similar to that of bupivacaine, though studies in vitro and in vivo have shown that ropivacaine is less cardiotoxic. Clinical data suggest that ropivacaine is clinically safer than bupivacaine [26].

Levobupivacaine and ropivacaine have a clinical profile similar to racemic bupivacaine, and the minimal differences that occurred between these three anesthetics are related mainly to a slightly different anesthetic efficacy, which decreases in the order: racemic bupivacaine > levobupivacaine > ropivacaine [49].

For levobupivacaine and ropivacaine, many clinical studies of two new stereoselective local anesthetics in the form of (*S*)-(−)-enantiomers have been conducted, examining their toxicology and clinical profiles: some differences were observed theoretically and experimentally, but the effects of these properties on clinical practice have not been proven [21,50,51]. Comparisons of the effect of bupivacaine, levobupivacaine, and ropivacaine on the central nervous system and on the heart have been the subject of many papers [52,53].

### 3.6. IQB-9302

**Scheme 6:**
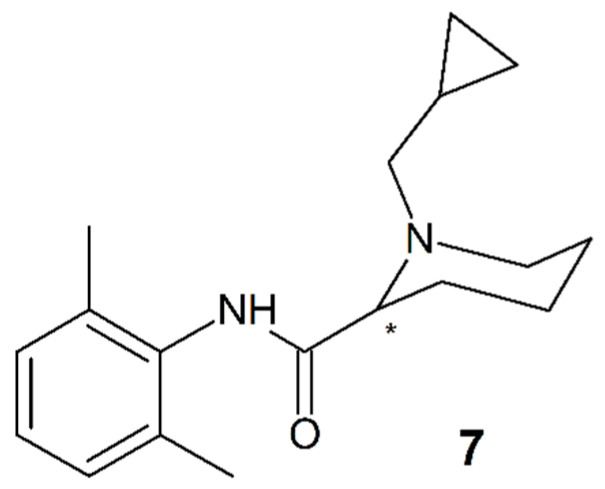
IQB-9302. (**7**).

This is a new amide-type anesthetic that has a cyclopropylmethyl chain bonded to piperidine nitrogen (**7**, Scheme 6), where it differs from bupivacaine and ropivacaine. It was found to block potassium channels Kv2.1 and Kv4.3 and the hERG channel similarly to bupivacaine. The hKv1.5 channels were found to be blocked in a manner 2.5 times weaker than with bupivacaine. The stereoselectivity of the blocking activity hKv1.5 channels resembles that of bupivacaine: the (*R*)-(+) enantiomer was the more active stereoiseomer (3.2 times higher activity compared to the (*S*)-(−) isomer of IQB-9302) [54]. The stereoselective effect seems to be connected to the different orientation of *N*-substituents in the (*R*)-(+) and (*S*)-(−) enantiomers, respectively. The authors also claimed that the length of the *N*-substituent in these local anesthetics and not its volume was crucial for the potency and degree of their stereoselective hKv1.5 channel block [46].

### 3.7. Articaine

**Scheme 7:**
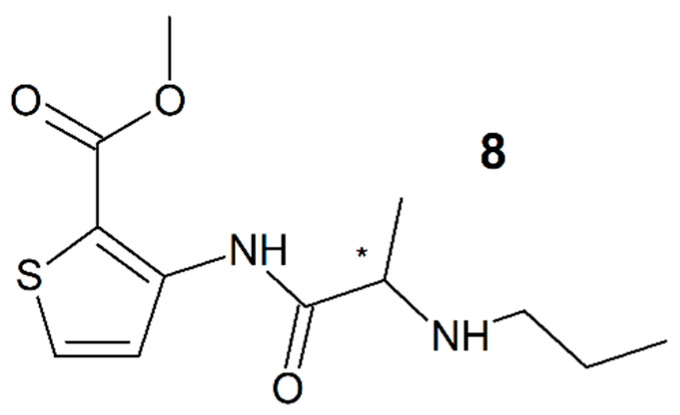
Articaine (**8**).

Articaine, *O*-methyl-4-methyl-3-[2-(propylamino)propanoylamino]tiophene-2-carboxylate (**8**, Scheme 7), a local anesthetic containing a substituted thiophene skeleton in its structure exhibits, in addition to the local anesthetic effect, a high degree of antimicrobial activity associated with the lipid interaction of prokaryotic membranes in both monolayers and bilayers [54].

The interactions of the racemic mixture of articaine, as well as the (*R*)- and (*S*)-enantiomer of articaine, with monolayers of glycerophospholipids and brain lipids were studied using the Langmuir monolayer technique. It was shown that the (*R*)-enantiomer better intercalated into the total lipid extract from pig brain (TLPBS) than the (*S*)-enantiomer [55,56].

### 3.8. Etidocaine

**Scheme 8:**
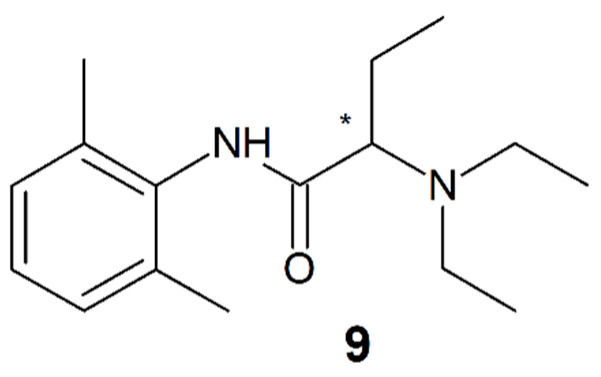
Etidocaine (**9**).

Etidocaine, *N*-(2,6-dimethylphenyl)-2-[ethyl(propyl)amino]butanamide (**9**, Scheme 8), contains a stereogenic center, where it differs from the structurally similar lidocaine. It is characterized by prolonged effect and a faster anesthesia onset [57,58,59]. Differences in the local anesthetic activity of the individual stereoisomers have not been published.

### 3.9. Carbizocaine

**Scheme 9:**
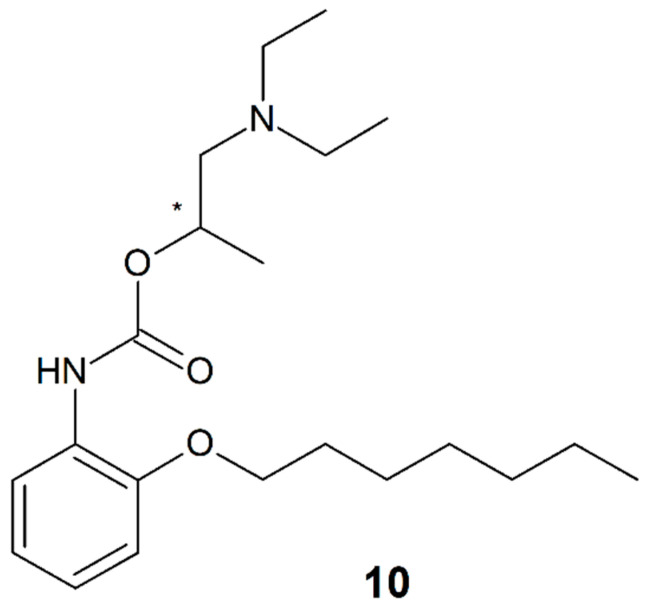
Carbizocaine (**10**).

The potential local anesthetic carbisocaine, (2-*N*,*N*-diethylaminopropan-1-yl)-(2-heptyloxyphenyl)carbamate (**10**, Scheme 9), was prepared from phenylcarbamic acid derivatives. During the testing of its enantiomers on the sciatic nerve of rats no significant differences were found between the anesthetic effect of the racemic form and of the individual enantiomers. Slightly lower local anesthetic activity was observed in the (−)-enantiomer [60].

### 3.10. Pentacaine

**Scheme 10:**
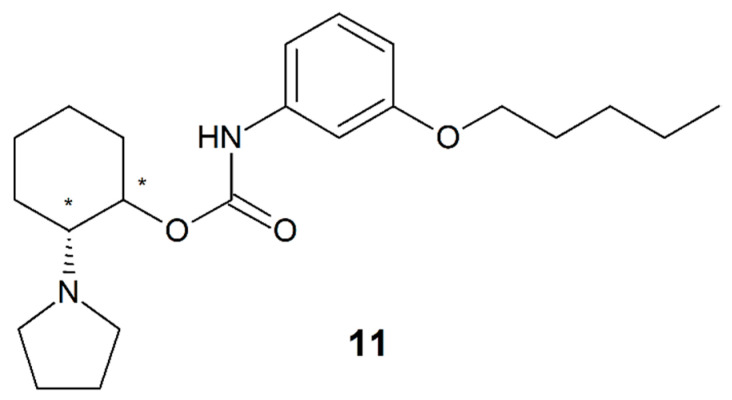
Pentacaine (11).

Pentacaine, *trans*-(±)-2-(pyrrolidine-1-yl)cyclohexyl(3-pentyloxyphenyl)carbamate (**11**, Scheme 10), also known as trapencaine, exhibits besides local anesthetic efficacy gastroprotective and antiulcerative activities [61,62,63]. On the rat models used, the antiulcer effect was more pronounced in the case of the (+)-*trans* enantiomer than in the (+)-*cis* enantiomer.

The effects of (±)-*trans*-2-(pyrrolidin-1-yl)cyclohexylester and its (+)-*trans* and (−)-*trans* enantiomers, and (±)-*cis*-2-(pyrrolidin-1-yl)cyclohexylester and its (+)-*cis* and (−)-*cis* enantiomers on length and number of gastric lesions induced by indomethacin and by 96% ethanol in rats were studied. With indomethacin the differences between (±)-*trans*-2-(pyrrolidin-1-yl)cyclohexylester and its (+)-*trans* and (−)-*trans* enantiomers were not significant. However, the best protective effect (around 80%) was achieved after pretreatment with the (+)-*trans* isomer. The least effective drug was (−)-*trans*-enantiomer. Similar results were obtained for lesion number. In ethanol-induced injury the greatest protective effect was observed after pretreatment with (+)-*trans*-enantiomer.

Activities of the *cis* isomers were only of minor significance in the indomethacin model of gastric injury. Both the lesion length and lesion number were greater in comparison with control values. Pretreatment with (−)-*cis* enantiomer even increased the damage. The differences between (+)-*cis* and (−)-*cis* enantiomers were also insignificant. In the indomethacin model the antiulcer effect was higher with the (+)-*trans* enantiomer when compared with the (+)-*cis* enantiomer. [64]

The absolute configuration of the stereoisomers has not been reported. Only data on the differences in antiulcer activities of the discrete enantiomers are available so far.

### 3.11. Fomocaine

**Scheme 11:**
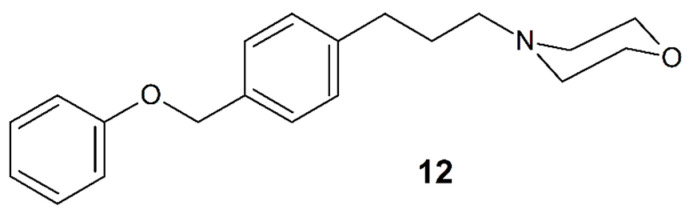
Fomocaine (**12**).

**Scheme 12:**
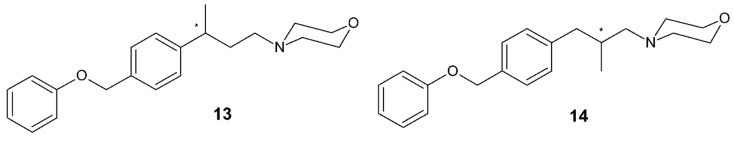
Chiral derivatives (+)-O/G5 (**13**) and (−)-O/G3 (**14**) of compound (**12**).

Based on changes in the activity of anilide-group stereoisomers, studies were conducted with stereoisomers of fomocaine derivatives from the base ether family. Fomocaine 4-[3-(4-(phenoxymethyl)phenyl)propyl]morpholine (**12**, Scheme 11), does not contain a stereocenter, but its two chiral derivatives (+)-O/G5 (**13**) and (−)-O/G3 (**14**) (depicted in Scheme 12) were compared with fomocaine and procaine. Surface local anesthetic activity in both isomers was lower than in procaine. (+)-O/G3 and (−)-O/G3 exhibited higher activity than fomocaine, but without practical application. Minor differences between the efficacy of the enantiomers of both O/G3 and O/G5 were found (these were more pronounced for O/G5), but this small distinction of effect is without practical consequences. Neither of the enantiomers of the two derivatives is more or less effective than the other, although in the case of O/G5 slight agonistic effects of both enantiomers could be shown. [65,66,67].

## 4. Stereoselectivity in the Pharmacokinetics of Local Anesthetics

Apart from their pharmacodynamic characteristics, many local anesthetics also differ in their pharmacokinetic properties. Of the individual phases of pharmacokinetics, the processes of absorption, distribution, and metabolism of individual local anesthetics have been monitored.

### 4.1. Bupivacaine (4)

Comparisons of the individual pharmacokinetic parameters on various models have been conducted mainly for bupivacaine. At plasma concentrations lower than 40 μM, (*R*)-(+)-bupivacaine exhibited higher affinity for plasma proteins (alpha-1-acid glycoprotein, AAG) than (*S*)-(−)- bupivacaine. At a concentration greater than 60 μM the opposite phenomenon was observed, with a higher concentration of unbound (*R*)-(+)-bupivacaine [68]. Martínez-Gómez [69] confirmed that (*S*)-bupivacaine exhibited a higher affinity for plasma proteins than its opposite enantiomer.

In adult volunteers, with bupivacaine there was a higher free fraction of (*R*)-(+)-enantiomer compared to (*S*)-(−)-enantiomer. The clearance of the unbound form of the (*R*)-enantiomer was somewhat lower than the clearance of the unbound form of the (*S*)-enantiomer, demonstrating that the effect of the unbound concentration of (*S*)-bupivacaine is lower than for (*R*)-bupivacaine [70,71].

In ovine models, the values of liver and total body clearance of (*R*)-bupivacaine were up to 30% higher than for the (*S*)-enantiomer of bupivacaine [72]. The distribution coefficient of (*R*)-bupivacaine was higher in the heart, brain, and other organs as compared to the distribution coefficient of the (*S*)-enantiomer [73]. In dog model, the pharmacological behavior of (*S*)-(−)-bupivacaine was similar to that of the racemate of bupivacaine, but the motor block attributed to (*S*)-(−)-bupivacaine lasted longer, which may be attributed to the racemate [74].

A study by Fawcett [75] showed that although bupivacaine pharmacokinetic parameters are significantly different, there is no evidence of chiral inversion after administration of (*S*)-bupivacaine. The urine of patients after administration of racemic bupivacaine by epidural infusion was observed to contain a greater amount of unchanged (*R*)-bupivacaine than its (*S*)-(−)-enantiomer. HPLC analysis of urinary metabolites in patients after epidural infusion of racemic bupivacaine confirmed that (*S*)-bupivacaine was more intensively metabolized than (*R*)-bupivacaine, with the predominant metabolic pathway being dealkylation [76].

The total and unbound concentrations of bupivacaine enantiomers in human plasma were reported [77]. The pharmacokinetics of bupivacaine were enantioselective, showing a lower plasma proportion of the (*R*)-enantiomer. (*R*)-bupivacaine also exhibited a higher unbound fraction (10.84%) than the (*S*)-isomer (6.29%).

### 4.2. Prilocaine (3)

The pharmacokinetics of the prilocaine enantiomers were evaluated after intravenous administration of racemates, and stereoselectivity was found. The difference in clearance is attributed to the resulting difference in internal metabolic clearance. The difference in the pharmacokinetics of prilocaine enantiomers is not essential for clinical practice [78]. The study of Herdevall et al. [79] stated that the toxicity of (*S*)-prilocaine was related to its metabolic conversion to o-toluidine.

The study of Copeland [80] monitored the clearance of individual enantiomers in sheep. The enantiomers (*R*)-bupivacaine and (*R*)-prilocaine exhibited greater clearance than their (*S*)-enantiomers. In the case of mepivacaine, clearance was not enantioselective.

### 4.3. Mepivacaine (2)

In the study of pharmacokinetics of this local anesthetic, differences in the optical rotation of the individual enantiomers were observed. In the case of bupivacaine and ropivacaine, (*S*) enantiomers showed a (−) rotation, while enantiomers (*R*) showed a (+) rotation. Due to the small structural change in the case of mepivacaine in comparison with these local anesthetics, different designations of rotation appear in many works, thus not allowing generalizations on individual stereoisomers in relation to their local anesthetic activity [24,34,81,82].

The higher plasma concentration values of (*S*)-(−)-mepivacaine were found to be caused by the smaller distribution volume and slower clearance [81]. Similarly, higher free fractions in volunteers were observed for (*R*)-(−)-mepivacaine in comparison with the (*S*)-(+)-enantiomer. Total plasma clearance and distribution volume for the (*R*)-(−)-isomer were two times greater compared to the (*S*)-(+)-mepivacaine [82]. Differences in these pharmacokinetic parameters of both enantiomers of mepivacaine and bupivacaine had previously been followed in ovine models [83].

### 4.4. Ropivacaine (6)

The plasma concentration of ropivacaine is dependent on the dose administered and the route of administration as well as on hemodynamic conditions. Ropivacaine as a chiral long-acting local anesthetic, similarly to levobupivacaine, was metabolized by *N*-dealkylation to 2’,6’-pipecoloxylidide (PPX). Through aromatic hydroxylation, hepatic cytochrome P450 3’-OH ropivacaine and 4’-OH ropivacaine were produced. PPX was the major metabolite in human liver microsomes. CYP3A4 cytochrome was involved in the formation of PPX and CYP1A2 cytochrome in the production of 3’-OH-ropivacaine in human liver microsomes [84].

**Figure 2:**
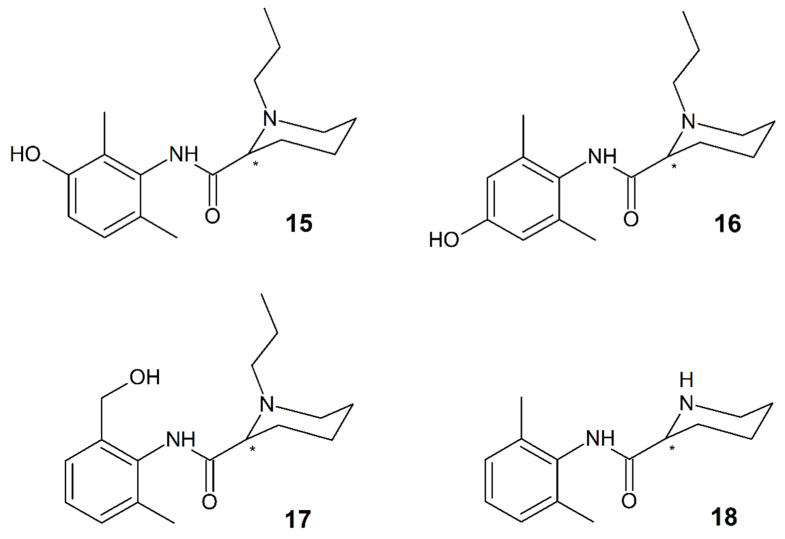
Selected metabolites of ropivacaine (**6**).

Ropivacaine is largely eliminated by extensive hepatic metabolism, with only 1% of the dose being eliminated unchanged in human urine. The four metabolites produced in human liver microsomes were identified as 3-OH-ropivacaine (**15**), 4-OH-ropivacaine (**16**), 2-OH-methyl-ropivacaine (**17**), and 2’,6’-pipecoloxylidide (**18**) (Figure 2) [85].

## 5. Stereoselective Analysis

The preparation of pure enantiomers, pharmacological/pharmacokinetic studies, and therapeutic evaluation of chiral drugs require the use of effective stereoselective analytical methods. In the enantioseparation of local anesthetics with a chiral centre various kinds of methodologies are used, the most common among them being HPLC on chiral columns. The methodologies are used in particular for enantioseparation or for the study of pharmacokinetic parameters.

### 5.1. High-Performance Liquid Chromatography

Chromatographic separation of cocaine and pseudococaine by liquid chromatography was performed using a chiral stationary phase (Supelcosil-LC-urea, Sigma-Aldrich Co., St. Louis, MO, USA) and acetonitrile as a mobile phase. The method can be used in forensic toxicology to investigate whether or not illegal cocaine samples come from a synthetic source [31].

Gatley [86] studied the behavior of individual enantiomers of cocaine with butyrylcholinesterase. It was found that synthetic (+)-cocaine was hydrolysed more than 2000 times faster than natural (−)-cocaine, which may be related to the pharmacological ineffectiveness of the (+)-isomer.

In the study of Siluveru [87], enantioselective separation of the four racemic local anesthetics bupivacaine, mepivacaine, prilocaine, and etidocaine was performed on cellulose tris 3,5-dimethylphenylcarbamate (Chiralcel OD / Daicel Corporation, Osaka, Japan) and cellulose tris benzoate (Chiralcel OJ / Daicel Corporation, Osaka, Japan). A baseline distribution with peak resolution R_s_ greater than 1.5 was achieved in bupivacaine and prilocaine on Chiralcel OD using a 99:1 *v*/*v* mobile phase and for etidocaine on Chiralcel OJ using hexane/ethanol in a ratio 98:2. Etidocaine and mepivacaine on Chiralcel OD gave only partial separation and the Rs values were lower than 0.81. In the separation, various protic and aprotic modifiers were studied.

These authors in later work studied the separation of mepivacaine on Pirkle brush (*S*)-*tert*-leucine, (*R*)-1-(α)-naftyl)ethylamine Sumichiral OA-4700 (Sumika Chemical Analysis Service, Ltd., Osaka, Japan) in human serum following isolation by solid phase extraction using the mobile phase hexane:ethylene dichloride:absolute methanol (85:10:5 *v*/*v*). Bupivacaine was selected as an internal standard [88].

The stereoselective HPLC for articaine and its metabolite articainic acid was studied using the Chiralcel OD and the Chirobiotic V (Sigma-Aldrich Co., St. Louis, MO, USA) columns. The Chirobiotic OD chiral column with cellulose tris-dimethylphenyl-carbamate and mobile phase hexane:propane-2-ol for articaine and hexane: ethanol for articainic acid produced zero line enantioseparation. Good enantioseparation was exhibited also by the chiral column Chirobiotic V, based on vancomycin with 5% acetonitrile in triethylammonium acetate [89].

**Scheme 13:**
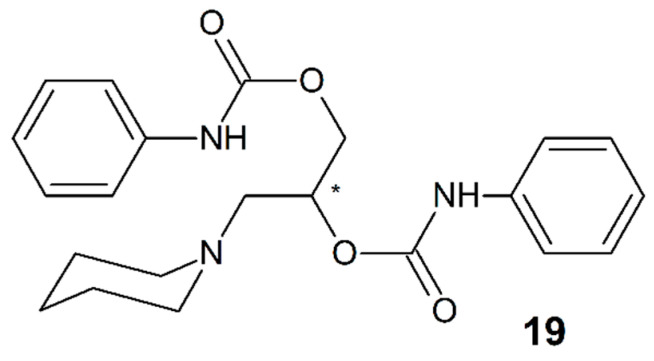
Diperodone enantiomer (**19**).

For the separation of diperodone enantiomers (**19**, Scheme 13), a chiral stationary phase based on teicoplanin with a polar organic mobile phase consisting of methanol/acetonitrile/acetic acid/trimethylamine 45:55:0.3:0.2, *v*/*v*/*v*/*v* was used. The elaborated method was suitable for determination of blood serum enantiomers at concentrations above 0.5 μg/mL. To study the degradation of diperodone enantiomers, an in vitro method was used, and experimental rate constants were calculated [90].

Enantioselection of diperodon on cyclodextrin/teicoplanin, teicoplanin aglycone and 3,5-dimethylphenylcarbamate cyclofructam 7, using acetonitrile/methanol/acetic acid/triethylamine 80/20/0.3/0.2 *v*/*v*/*v*/*v*, was successful only on chiral columns based on teicoplanin (T and TAG / Sigma-Aldrich Co., St. Louis, MO, USA) [91].

The separation of the potential carbamate-type local anesthetic of carbisocaine into individual enantiomers was performed on a β-cyclodextrin chiral stationary column using a mobile phase consisting of 5% acetonitrile in water and 0.01% triethylamine. Enantioseparation depended mainly on the quantity of acetonitrile and the elution order of the individual enantiomers was (*R*)-(−) before (*S*)-(+) [85]. Enantioseparation of carbisocaine on these columns was unsuccessful [92].

**Figure 3:**
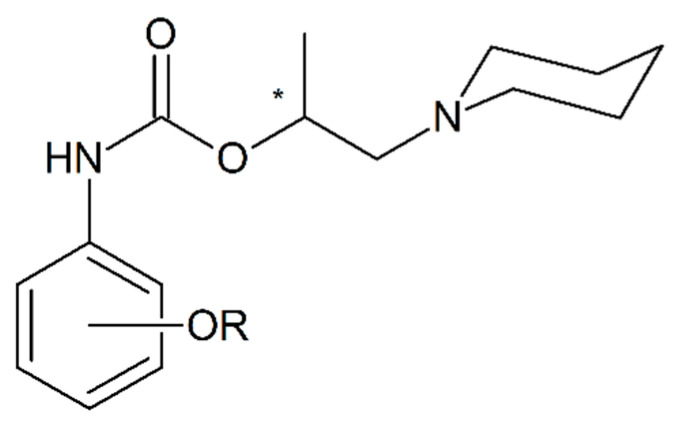
Derivatives of phenylcarbamic acid.

An extensive study of substituted phenylcarbamic acid esters (Figure 3) was carried out on several chiral columns.

For separating enantiomers of the 1-methoxymethyl, 1-ethoxymethyl, 1-propoxymethyl-2-(1-pyrrolidinyl), (1-piperidino), (1-perhydroazepinyl)etyl-, and 2-alkoxyphenylcarbamates, a column on a cyclodextrin base was used. The effects of the structures of these compounds and the effect of temperature on enantioseparation were studied. A decrease in temperature caused an increase in the retention factors of the examined compounds and in the resolution values of the individual enantiomers [93]. The enantioseparation mechanism was studied for a similar type of compound [94].

For separating and determining the enantiomers of alkoxysubstituted phenylcarbamic acid esters in the blood serum, columns filled with vancomycin bound on the C18 achiral column and β-cyclodextrin were used. The method involved off-line separation of the racemate in the reversed-phase stationary phase and separation of the enantiomers on the chiral stationary phase. The detection limit was 1.0 μg/mL for the vancomycin column and 10.0 μg/mL for the column with β-cyclodextrin in a standard solution. It was found that the (*R*)-(−)- and (*S*)-(+)-enantiomeric rate constants did not differ significantly [95].

Another study used the teicoplanin chiral stationary phase and teicoplanin chiral stationary phase without additional carbohydrate groups (Chirobiotic teicoplanin aglycone (TAG) / Sigma-Aldrich Co., St. Louis, MO, USA). The effects of sugar units, the concentration of ionic modifiers (diethylamine), the position and number of carbon atoms in the alkoxy chain, and the composition of the mobile phase were evaluated. Better separation, shown by higher R_ij_ (relative retention) enantiomer values, was achieved using a Chirobiotic TAG column (Sigma-Aldrich Co., St. Louis, MO, USA) [96].

In the work of Ďungelova et al. [97], the conditions for the enantioseparation of the alkoxysubstituted phenylcarbamic acid esters on the vancomycin-based chiral column were investigated. Different factors were investigated, including the type and concentration of ionic modifiers in the mobile phase, and the mechanism of separation interactions was discussed.

As part of a pharmacokinetic study, the enantiomers of bupivacaine in human plasma were separated on a Chirex 3020 chiral stationary phase column (Phenomenex Inc., Torrance, CA, USA) [77]. The lower limit of quantification was 0.25 ng of each enantiomer/mL of plasma as the total concentration and 0.125 ng of each enantiomer/mL of plasma as the unbound concentration.

The HPLC analyses of chiral compounds with local anesthetic activity are summarized in Table 2.

### 5.2. Capillary Electrophoresis

In addition to HPLC, chiral analysis of local anesthetics often employs capillary electrophoresis, using various chiral selectors.

For the local anesthetics mepivacaine, ropivacaine, bupivacaine, and prilocaine, their interaction with heptakis (2,6-di-*O*-methyl-β-cyclodextrin) and triethylamine added to the base electrolyte to improve enantioseparation was studied [98].

In the work of Siluveru and Steward concerning the enantioseparation of racemic prilocaine, various kinds of β-cyclodextrin were used, of which only heptakis (2,6-di-*O*-methyl β-cyclodextrin) with the mobile phase hexadecyltrimethylammonium bromide in phosphate buffer proved to be suitable. The developed method for the quantification of prilocaine in human serum was compared with HPLC analysis in the work [99].

In one report [100], an on-line combination of capillary electrophoresis with micro-electrospray mass spectrometry was used for the first time in the enantioseparation of bupivacaine and ropivacaine. The chiral selector methyl-β-cyclodextrin was introduced into the selector with the aid of polyacrylonitrile in order to minimize electro-osmotic flow.

Non-covalent complexes between three different cyclodextrins (CD) and six local anaesthetics were studied using capillary electrophoresis (CE) and electrospray ionisation mass spectrometry (ESI-MS) [101].

The use of methylcyclodextrin in studying the enantiomeric purity of (S)-ropivacaine was the subject of the report [102].

Concurrent enantioseparation of selected anesthetics was studied by capillary electrophoresis in the presence of three different negatively charged cyclodextrins (CDs). Of the tested chiral selectors, carboxymethyl, sulfobutyl ether, and sulfated-β-CD appeared to be the most effective in achieving enantiomeric resolution of the investigated compounds. Optimal electrophoretic conditions for stereoselective analysis of the studied anesthetics were obtained using a polyvinyl alcohol-coated capillary (total length 48.5 cm × 50 μm ID), 50 mM Tris-phosphate buffer at pH 2.5 containing 6 mg mL^−1^ sulfated β-CD, a voltage of 30 kV, and a temperature of 30 °C [103].

In rabbit serum and pharmaceutical injections, sulfobutyl ether-cyclodextrin was also suitably used for the enantioseparation of bupivacaine [104].

Bupivacaine was also separated using a partial filling technique with the human serum albumin as the chiral selector and the cationic surfactant cetyltrimethylammonium bromide [105].

Amini et al. [106], in separating local anesthetics, applied micellar electrokinetic chromatography with taurodeoxycholate as a chiral selector and surfactant. Enantioseparation was dependent on the concentration of taurodeoxycholate.

The conditions of the CE separations described above are summarized in Table 3.

## 6. Conclusions

The presented work provided an overview of local anesthetics, which due to different stereochemistry vary in local anesthetic activity, pharmacokinetic properties, and toxicity. The overview was focused on cocaine, bupivacaine, mepivacaine, prilocaine, ropivacaine, IQB-9302, articaine, etidocaine, carbisocaine, and the stereoisomer of fomocaine. In many of these, the data on the local anesthetic activity of individual (*R*) and (*S*) stereoisomers were very incomplete, with insufficiently accurate characterization of the optical rotation of individual enantiomers, particularly in the pharmacokinetics of mepivacaine. In clinical practice only levobupivacaine and ropivacaine are used as (*S*)-(−)-stereoisomers. The chiral local anesthetics are summarized in Table 4.

Of the individual phases of pharmacokinetics, the processes of absorption, distribution, and metabolism of individual local anesthetics were monitored. Changes in individual enantiomers were studied for bupivacaine, mepivacaine, prilocaine, and ropivacaine.

For enantioselective separation of racemates into individual enantiomers, chromatographic and electrophoretic methods were used. In the case of HPLC, direct methods used various chiral phases based on cyclodextrins, macrocyclic antibiotics (vancomycin, teicoplanin) on substituted carbamates of cellulose, and amylose, many of which are the basis of chiral selectors in direct methods in capillary electrophoresis.

It can be concluded that the potential for the use of enantiomerically pure local anesthetics is not yet fully realized, despite their advantageous properties in terms of higher activity and lower toxicity. Much work is still needed to clarify the differences in the pharmacological and toxicological behavior of racemic anesthetics and their respective enantiomers.

## Figures and Tables

**Table 1:** Physical-chemical/pharmacokinetic properties and toxicity of some local anesthetics.

INN ^1^	pK_a_	Partition Coeffic. ^2^	Solubility pH 7.4	Protein Binding (%)	Half-Life	Speed on Onset	Duration	Toxicity
Mepivacaine	7.6	21	Moderate	78	2.0–3.0 h	Fast	Intermediate	Low
Prilocaine	7.9	25	Moderate	6	1.25h	Slow	Intermediate	Low
Bupivacaine	8.1	346	Very good	95	2.7–3.5 h	Slow	Long	High
Ropivacaine	8.1	115	Very good	94	1.8 h	Slow	Long	Intermediate
Articaine	7.8	32	Moderate	95	0.5 h	Very fast	Long	Low
Etidocaine	7.7	800	Very good	94	2.7 h	Fast	Intermediate	Intermediate
Lidocaine	7.9	43	Moderate	77	1.5–1.8 h	Fast	Intermediate	Low
Procaine	8.9	1.7	Low	6	30–50 s	Slow	Short	Low
Tetracaine	8.2	221	Very good	76	2–4 min	Fast	Short	Intermediate

^1^ international nonproprietary name, ^2^ octanol—water partition coefficient.

**Table 2:** Chiral stationary phases and mobile phases used to separate local anesthetics by HPLC.

Chiral Stationary Phase	Mobile Phase	Chiral Local Anesthetic	Ref.
Supelcosil LC-urea	Acetonitrile	Cocaine	[31]
Chiracel OD	Hexane/ethanol 99:1 *v*/*v*	Bupivacaine, mepivacaine, prilocaine	[87]
Chiracel OD	Hexane/propan-2-ol	Articaine	[89]
Chiracel OJ	Hexane/ethanol 98:2 *v*/*v*	Etidocaine	[87]
Sumichiral OA-4700	Hexane/dichloroethane/methanol 85:10:5 *v*/*v*/*v*	Prilocaine	[88]
Chirobiotic V	5% Acetonitrile in triethylammonium acetate	Articaine	[89]
Chirobiotic T	Methanol/acetonitrile/acetic acid/triethylamine 45:55:0.3:0.2 *v*/*v*/*v*/*v*	Diperodon, alkoxyphenylcarbamates	[90]
Chirobiotic TAG	Acetonitrile/methanol/acetic acid/triethylamine 80:20:0.3:0.2 *v*/*v*/*v*/*v*	Diperodon	[91]
β-CD	% Acetonitrile in water and 0.01% triethylamine	Carbisocaine	[92]
β-CD, Chirobiotic TAG	Methanol/acetic acid/diethylamine 100 *v*/17.49 mmol/0–9.57 mmol/L	Alkoxyphenylcarbamates	[96]
Chirex 3020	95% Hexane/ethanol (80:20, *v*/*v*)	Bupivacaine	[76]

**Table 3:** Chiral selectors and background electrolyte used in capillary electrophoresis of local anesthetics.

Chiral Selector	Part of Background Electrolyte	Chiral Local Anesthetic	Reference
Heptakis(2,6-di-*O*-methyl- ß-CD)	Triethanolamine	Mepivacaine, ropivacaine, bupivacaine	[98]
Heptakis(2,6-di-*O*-methyl- ß-CD)	Phosphate buffer	Prilocaine	[99]
Methyl-ß-CD	Phosphate buffer	Bupivacaine, ropivacaine	[100]
methyl-ß-CD	Acetate buffer	Bupivacaine, ropivacaine	[102]
Carboxymethyl, sulfobutyl ether and sulfonated-β-CD	Tris–phosphate buffer	Bupivacaine, mepivacaine, prilocaine	[103]
Sulfobutyl ether	Acetate buffer	Bupivacaine	[104]

**Table 4:** Selected chiral local anesthetics.

Local Anesthetic	Total Number of Stereoisomers	Most Active Isomer	More Toxic Isomer	Used as
Cocaine (**1**)	8	(1*S*,2*S*,3*R*,5*R*)-(+)	(1*R*,2*R*,3*S*,5*S*)-(−)	(1*R*,2*R*,3*S*,5*S*)-(−)
Mepivacaine (**2**)	2	(*S*)-(−)	NA	Racemate
Prilocaine (**3**)	2	Comparable	(*R*)-(−)	Racemate
Bupivacaine (**4**)	2	Comparable	(*R*)-(+)	(*S*)-(−)
Ropivacaine (**6**)	2	NA	(*R*)-(+)	(*S*)-(−)
Articaine (**8**)	2	NA	NA	Racemate
IQB-9302 (**7**)	2	NA	NA	Racemate
Etidocaine (**9**)	2	NA	NA	Racemate
Carbisocaine (**10**)	2	Comparable	NA	Racemate
Pentacaine (**11**)	4	NA	NA	Racemate
OG/5 (**13**)	2	NA	NA	Racemate
OG/3 (**14**)	2	NA	NA	Racemate

NA = sufficient data not available or no definite conclusions possible; comparable = differences in the activity of the respective enantiomers are insignificant.
